# Model-based meta-analysis of individual patient data for the characterization of intravenous 5-fluorouracil population pharmacokinetics

**DOI:** 10.1007/s00280-026-04885-x

**Published:** 2026-06-12

**Authors:** Myriam Briki, Paul Thoueille, Markus Joerger, Etienne Chatelut, Marylise Sterle, Antonin Schmitt, Anna Dorothea Wagner, Thierry Buclin, Sandro Carrara, Monia Guidi

**Affiliations:** 1 Service of Clinical Pharmacology, Department of Medicine, Lausanne University Hospital and University of Lausanne, Lausanne, Switzerland; 2 Bio/CMOS Interfaces Laboratory, Department of Engineering, Swiss Federal Institute of Technology in Lausanne - EPFL, Lausanne, Switzerland; 3 Laboratory of Clinical Pharmacology, Department of Laboratory Medicine and Pathology, Lausanne University Hospital and University of Lausanne, Lausanne, Switzerland; 4 Department of Oncology and Hematology, Cantonal Hospital St. Gallen, St. Gallen, Switzerland; 5 IUCT-Oncopole, University of Toulouse, Oncopole Claudius Regaud, Toulouse, INSERM UMR1037 France; 6 Université Bourgogne Europe, Centre Georges-François Leclerc, Service Pharmacie, INSERM, CTM UMR 1231, TiRECs/STARTER-BFC, Dijon, France; 7 Department of Oncology, Division of Medical Oncology, Lausanne University Hospital and University of Lausanne, Lausanne, Switzerland; 8 Centre for Research and Innovation in Clinical Pharmaceutical Sciences, Lausanne University Hospital and University of Lausanne, Lausanne, Switzerland

**Keywords:** Fluorouracil, Pharmacokinetics, Therapeutic drug monitoring, Model-informed precision dosing, Personalised care, Oncology

## Abstract

**Purpose:**

The pharmacokinetics (PK) of 5-fluorouracil (5-FU) has been thoroughly studied over the past decades. Using an individual patient data model-based meta-analysis (IPD-MBMA) approach, this study aims to build a generalized model by combining IPD from multiple studies, investigating the role of lean body mass (LBM) in 5-FU PK, and exploring a model informed precision dosing (MIPD)-based approach for refining its therapeutic drug monitoring (TDM).

**Methods:**

Raw PK data from three 5-FU PK studies (726 patients, 2,332 plasma concentrations) were harmonized and grouped into one homogeneous dataset, preserving traceability to the original data source. An overall population PK (popPK) model for intravenous 5-FU was developed using nonlinear mixed-effects modeling (NONMEM).

**Results:**

A two-compartment model with both saturable (Michaelis-Menten elimination; V_max_, K_m_) and linear elimination (CL) best described 5-FU disposition, with a stratified error model to capture between-study differences. Body-surface area (BSA) significantly impacted CL, and V_max_ was 15% lower in women and decreased with age. In a parallel analysis, the sex effect on V_max_ was translated into an LBM effect, with metabolic capacity increasing with LBM. Based on a 12-cycle simulation, after 4 cycles of TDM-based dose adjustment, 95% of the patient should be optimally exposed in the context of colorectal cancer (area under the curve, AUC 20–29 mg∙h/L). A virtual MIPD-based approach predicted higher target attainment after one adjustment (70 − 68%) compared to classic TDM predictions (49–59%) in men and women, respectively. Finally, the administration of a bolus before the continuous infusion of 5-FU is not expected to materially affect its subsequent continuous infusion AUC.

**Conclusion:**

Aggregating IPD enables building more generalizable popPK models while accommodating study-specific residual variability; broader raw-data sharing would strengthen current evidence and reveal remaining gaps. For 5-FU, BSA dosing leaves substantial interpatient variability, so LBM-based and model-informed strategies, including *a priori* dosing and MIPD-assisted TDM, warrant prospective evaluation.

**Supplementary Information:**

The online version contains supplementary material available at https://doi.org/10.1007/s00280-026-04885-x.

## Introduction

In cancer chemotherapy, the maximum-tolerated dose (MTD) approach has long dictated drug dosing, driven by the certitude that higher doses increase the chances of eradicating cancer cells, but it often ignores severe delayed toxicities [1, 2]. Body surface area (BSA)-based dosing emerged from MTD normalization in phase I trials and became standard across antineoplastics—yet its rationale has been debated for decades [3–6]. Indeed, several pitfalls are identified in this practice. Firstly, BSA is calculated, not measured, and studies have shown that the true value may be underestimated: BSA-based dosing therefore seems precise yet lacks accuracy [7]. Secondly, BSA is a metric that involves height and weight and overlooks body-composition, the latter being determinant in chemotherapy, as cytotoxic agents mostly distribute in lean tissues and their clearance correlates best with the metabolically active part of the body mass [8]. This last point raises the question of whether BSA is essentially an indirect estimator of fat-free mass (FFM), or of its surrogate lean body-mass (LBM), which would represent more appropriate markers for individualizing drug dosages. Recent investigations have shown that, in the context of adjuvant oxaliplatin chemotherapy, for example, LBM-based dosing significantly decreases peripheral neurotoxicity without altering treatment efficacy [9]. Improving the dosing of chemotherapies beyond BSA-based adjustment may be feasible and is definitely worth investigating.

In parallel, growing interest in pharmacokinetic-pharmacodynamic (PK-PD) relationships has drawn attention to the link between drug exposure and both therapeutic efficacy and toxicity [10]. PK-PD studies indeed allow the identification of a therapeutic window in which both sufficient efficacy is achieved and toxicity is minimized, to enable meaningful dose adaptation based on the measured drug exposure marker. This ensures that the optimal amount of drug is circulating—a practice referred to as therapeutic drug monitoring (TDM). A prime example is the antimetabolite 5-fluorouracil (5-FU), for which numerous studies have demonstrated clear PK-PD relationships, making TDM increasingly recommended to refine the use of this potent cytotoxic agent, with evidence supporting its clinical benefit [11–13]. 

Evaluating the patient’s dihydropyrimidine dehydrogenase (DPD) enzyme status through genotyping or phenotyping (i.e., direct measure of enzymatic activity or measurement of endogenous uracil) helps prevent 5-FU overexposure and related toxicity [14]. Yet, despite clinical benefit, economic interests and reported feasibility, DPD status assessment is not systematically performed: oncologists’ awareness remains limited [15], and many rare genetic variants of DPD are not identified to date by conventional pharmacogenetic tests [16]. Beyond this, DPD genotype does not explain all variability in 5-FU clearance [17]. Therefore, it is far from certain that DPD genotyping or phenotyping could entirely substitute TDM in the precision dosing of 5-FU [18]; its role might be at best complementary, in particular for determining initial dosing. Together with reliable analytical techniques and available dose-adjustment guidelines, all the elements are in place for making the precision dosing of 5-FU both robust and convenient [19]. 

5-FU TDM is a particularly illustrative case of precision medicine applied to a drug with high interindividual PK variability. The systemic exposure (area under the concentration–time curve, AUC) is the PK endpoint: in the context of colorectal cancer treatment, for instance, an AUC window of 20–29 mg∙h/L is targeted [11]. Theoretically, 5-FU’s short elimination half-life (8–10 min), coupled with a prolonged infusion, should enable a straightforward AUC calculation by multiplying infusion duration by steady-state concentration [11]. However, despite many advantages, 5-FU TDM as currently practiced still faces some limitations. The concentration plateau of 5-FU, typically maintained during a 46-hour infusion either via an elastomeric or electronic pump, needs up to 18–26 h after infusion start to stabilize, despite its short half-life [11]. Indeed, 5-FU accumulates over time during the continuous infusion due to its inhibitory mechanism on its own metabolic pathway through the DPD enzyme; enough time is therefore needed for the concentration to stabilize [11]. In addition, current typical dosages of 5-FU comprise a 2-week rest period between infusions, meaning that dose adjustment can occur at the earliest for the next cycle rather than immediately. Moreover, the infusion should ideally run over the same hours of the day, given the known circadian variations in 5-FU metabolism [20, 21]. Finally, the usual method of calculating exposure does not account for whether a bolus is administered before the continuous intravenous (iv) infusion, leaving it uncertain how much the bolus might influence overall exposure. Any further insights that help identify patients at risk of overdosing or underdosing would therefore be highly valuable. These limitations indicate that 5-FU TDM might benefit from the support of a model-informed precision dosing (MIPD) approach, taking advantage of prior knowledge on its PK characteristics among patients to optimize its dosage adjustment according to measured circulating concentrations.

Population pharmacokinetic (popPK) analyses have been key in understanding the substantial interindividual variability (IIV) in 5-FU disposition, some of which can be partly explained by patient-specific covariates. Yet, while popPK studies can reveal and interpret variability in a specific cohort, their generalizability to other patient populations may not always be possible. The true robustness of a model is thus best confirmed through external validation—an ideal step rarely feasible due to the difficulty of accessing data from other populations [22]. 

Given the wealth of existing popPK models for 5-FU, a promising way to group and synthesize the entire information is through a model-based meta-analysis, ideally using individual patient data (IPD-MBMA) [23]. This approach allows different datasets to be aggregated into a unified meta-database, while retaining study identifiers to account for between-study differences. The robustness of this approach lies in the increased sample size as well as in the diversity of patient populations. The IPD-MBMA approach yet raises a few challenges: indeed, since the merged datasets come from different studies, the documented patient characteristics may not be the same, the analytical methods may differ, the 5-FU administration protocols, the ancillary treatments may be heterogeneous, etc. All these differences need to be kept in mind during the analysis, and ways to harmonize the data with as much precision as possible need to be considered.

In this study, we aimed to provide an insight into the power of joining multiple study populations together to perform IPD-MBMA, through the elaboration of a popPK meta-model for iv 5-FU. Our objective was to define predictors of 5-FU PK variability based on a large and representative dataset to inform an MIPD approach supporting 5-FU TDM. At the same time, we attempted to determine the respective importance of BSA and LBM in the disposition of 5-FU. We finally used this model to predict the effect of TDM-based dose adjustment on target AUC attainment throughout 5-FU cycles, as well as the impact of the bolus schedule on the resulting AUC calculated in clinical practice.

## Materials and methods

### Data acquisition

A thorough literature search was performed to identify popPK-compatible data for iv 5-FU model building from published articles. The criteria of inclusion were the following: study performed on humans, 5-FU concentrations measured after iv infusion, documented administered doses (schedules including amount and duration of infusion/bolus), and documented 5-FU samplings. The articles were collected using the prompt [fluorouracil] AND [population pharmacokinetics] in PubMed. Corresponding authors were contacted based on information available in the articles and asked them to share the individual patient data used in their analysis, as well as possible unpublished data if willing.

## PopPK analysis

The R software (version 4.2.1) was used to manage, clean, and harmonize the data of the obtained datasets, as well as for diagnostic plot visualization. Study-level identifiers (SID) as well as individual identifiers were attributed, time after dose was calculated in hours, doses and measured concentrations were harmonized to a respective common unit in mg and mg/L. The administration rate was therefore expressed in mg/h.

The nonlinear mixed effect modeling software NONMEM (version 7.6.0), assisted by Pirana workbench (version 2.9.3) and PsN (version 5.3.1), was used to develop the 5-FU meta-model and perform the simulations. Models were specified using ordinary differential equations (ADVAN13 library), and the first-order conditional method with interaction was employed (FOCEI). Of all measurements, 0.6% were below the lower limit of quantification (LLOQ) and remained simply censored in our dataset (M1 method) [24]. 

## Base model

The base model was built using a stepwise approach to identify the appropriate elimination process (linear, Michaelis-Menten, or combined), the number of compartments, the inclusion of IIV on PK parameters, and the error model for residual unexplained variability (RUV). For RUV, a single error model was first tested, and separate error models per study were then evaluated. Errors that were similar between studies were grouped if statistically advantageous (see model evaluation and selection). IIV of parameters was modeled using a log-normal distribution for the PK parameters and was expressed as coefficient of variation (CV%), using the transformation $\:\sqrt{\left({e}^{{\omega\:}^{2}}-1\right)}$, where ω^2^ is the variance of the random effect associated with the PK parameter. Inclusion of IIV on parameters was performed sequentially. Additional between- and within-study variabilities on PK parameters were tested as well (LEVEL item in NONMEM [25]).

## Covariate analyses

Using univariate and subsequent multivariate forward insertion and backward deletion procedures (stepwise selection process [26]), the following covariates were tested for significance on the base model parameters for which IIV was retained: sex, age, bodyweight (BW), height (HT), BSA (estimated with the Mosteller formula $\:BSA=\sqrt{\frac{HT\cdot\:BW}{3600}}$) [59], estimated glomerular filtration rate (eGFR, estimated using the CKD-EPI 2021 serum creatinine formula without ethnicity [60]), and DPD metabolic profile. Concerning the latter, we employed a categorical approach for phenotype data, as there was no standardized metric across the different studies (DPD activity, uracil concentration), and one dataset presented non-systematic reporting. Consequently, we divided patients into two binary categories, namely, potentially slow metabolizers versus others, using the cutoffs of 150 pmol/min/mg [27] for DPD activity and 16 ng/mL [28] for uracil concentration. Genotypic data was not investigated due to information scarcity in the dataset. For BW and BSA, allometric testing was applied, and a linear relationship was investigated for age and HT. Categorical testing was applied for sex and DPD metabolic profile. Regarding eGFR, both linear and sigmoid relationships were investigated. The covariate analysis was performed both with and without considering the allometric effect of LBM (estimated using Janmahasatian formula [29]):

$$\begin{aligned}\:&{LBM}_{men}=\:\frac{9270\cdot\:BW}{6680+216\cdot\:\frac{BW}{{HT}^{2}}}\:,\:\\&{LBM}_{women}=\:\frac{9270\cdot\:BW}{8780+244\cdot\:\frac{BW}{{HT}^{2}}}\end{aligned}$$

## Model evaluation and selection

The difference in the objective function values (ΔOFV) was used to compare hierarchical models in both base model development and forward covariate insertion with a significance threshold α = 0.05, i.e. ΔOFV ≤ -3.84 for the addition of one parameter. Backward deletion procedures were performed with a significance level of α = 0.01, i.e. ΔOFV ≥ 6.63 for the removal of one covariate. To compare non-hierarchical models, the difference in the Bayesian information criterion (BIC) values was used (ΔBIC < -2). The relative standard error (RSE) was used to evaluate the accuracy of the PK parameters, IIV, and covariate effects estimates. Furthermore, standard goodness-of-fit diagnostic plots, model shrinkage as well as distribution of random effects on parameters (ηs, evaluating IIV) and residuals (evaluating RUV) were employed to guide the final model selection (supplementary material).

Prediction-corrected visual predictive checks (pcVPC) were conducted to assess the final model’s predictive performance by comparing the 5th, 50th, and 95th prediction-corrected percentiles of observed and predicted data. Additionally, the model’s reliability was examined using a nonparametric bootstrap procedure with 2,000 replicates, which enabled comparison of the original parameter estimates with the bootstrap median values and their 95% confidence intervals (CI_95%_).

### Simulations

We performed simulations based on the final model, including RUV, in order to evaluate the evolution of target AUC attainment throughout 5-FU cycles after dose adjustment. A virtual population of 5,000 women and 5,000 men per SID (total of 30,000 virtual patients) was created based on the anthropometric distributions observed in the studied population. Specifically, we took the median characteristics for each sex, along with their observed standard deviations, and applied a normal distribution with a CI_95%_ to reflect the variability observed in our dataset. Observed correlations between height and weight were maintained, and subsequent BSA distributions matched the observed real-life distributions. We then simulated a 400 mg/m^2^ bolus administered over 10 min followed by a 46-hour continuous infusion of 2,400 mg/m^2^ 5-FU given 30 min after the bolus. This type of regimen is usually administered for up to 12 cycles, with 2-week breaks between cycles. We thus simulated the 12 cycles, each subsequent cycle benefiting from a dosage adjustment based on a TDM measurement made during the previous cycle.

AUC was calculated as the product of infusion duration (46 h) by the “observed” concentration (incl. RUV, further mentioned as Y = IPRED + RUV) simulated at 24.5 h, i.e. 24 h after the start of the continuous infusion. Dose adjustment for cycle *n* + 1 was based on the AUC calculated at cycle *n*, using the dose adaptation grid currently proposed (Table 1) [11]. Exceptional negative DVs, which result from the normal distribution of the additive error term (ε), were ignored.

**Table 1:** Dose adaptation grid for 5-FU

Determined AUC (mg∙h/L)	Adjustment for the next dose (*n* + 1)
> 40	30% dose reduction relative to the dose of cycle n
37–39	25% dose reduction relative to the dose of cycle n
34–36	20% dose reduction relative to the dose of cycle n
30–33	10% dose reduction relative to the dose of cycle n
20–29	No adjustment needed
17–19	10% dose increase relative to the dose of cycle n
14–16	20% dose increase relative to the dose of cycle n
8–13	25% dose increase relative to the dose of cycle n
< 8	30% dose increase relative to the dose of cycle n

Proposed dose adaptation grid from Lemaitre et al. (2018). Assessment of exposure AUC (mg∙h/L) = concentration (mg/L) × duration of infusion (h)

Next, we evaluated the impact of MIPD on AUC target attainment through our virtual TDM approach. From the first simulated cycle in our previous analysis, we extracted the individual predictions including RUV (Y = IPRED + RUV) at 24.5 h to mimic a real-life sampling scenario. Based on this unique “observed” concentration per patient, we used NONMEM to obtain maximum likelihood *post hoc* individual patient PK parameter estimates, also known as empirical Bayes estimates (MAXEVAL = 0 in the NONMEM EST item). Then, in a second step, using these estimates, we predicted a full PK curve for each patient to calculate IPRED_24.5 h_ and select the right dosage adjustment using the following multiplying factor *f* (details in the Supplementary Material):

$$\:f=\:\frac{{CL}_{i}\cdot\:{C}_{target}+\frac{{V}_{max,i}\cdot\:{C}_{target}}{{K}_{m,i}+{C}_{target}}}{{CL}_{i}\cdot\:{C}_{pred}+\frac{{V}_{max,i}\cdot\:{C}_{pred}}{{K}_{m,i}+{C}_{pred}}}$$

Where CL_i_, V_max, i_ and K_m, i_ are individual *post hoc* PK parameters estimates, C_target_ is AUC_target_/infusion time (here, 24 mg∙h∙L^− 1^ / 46 h, i.e. 0.52 mg/L), and C_pred_ is IPRED_24.5 h_. We compared the performance of this MIPD-like approach with the classic TDM approach - the latter only using the adaptation grid based on the previous cycle TDM result - calculating the percentages of target AUC attainment for each method. AUC was calculated as IPRED_24.5 h_ times 46 h, after checking that this led to results identical to those of a through numerical integration.

We additionally ran two other simulations on the same virtual population, with no bolus at all, a bolus given over 10 min with a 30 min wait before the continuous infusion, or the bolus administered over two minutes right before the start of the continuous infusion to evaluate the impact of bolus schedule on clinically assessed AUC.

## Results

### Data

Out of 16 identified studies meeting our criteria for inclusion, it was possible to obtain three independent datasets, with two published datasets from Terret et al. (2001) [30] and Mueller et al. (2013) [31], and one unpublished dataset containing retrospective routine care data from the Dijon Clinical Cancer Center, identified as SID1, SID2, and SID3, respectively, throughout this study. SID1 and SID2 collected written informed consent for all patients in their study. For SID3, patients were informed about secondary use of their data for research purposes and were included unless they objected (non-opposition), in accordance with French regulations. Details regarding available information and how they were harmonized are available in Table S1.

SID1 involves rich PK profiles of 21 patients, with a total of 159 measurements. In SID2, 31 patients underwent rich PK sampling as well, with a total of 329 samples, among which one patient was removed from our analysis (see section below). The last dataset SID3 includes a considerably larger population after data cleaning, with 675 patients and 1850 measurements, and rich PK sampling was performed for 21 of these patients. Although patients came from different studies, their characteristics are not drastically heterogeneous overall (Table 2). The analytical methods used to quantify 5-FU differed across studies, resulting in different LLOQs (Table 2). Values for LLOQ are comparable between SID1 and SID3 (0.05 mg/L and 0.03 mg/L, respectively), while SID2 allows lower concentrations to be quantified (LLOQ = 0.013 mg/L) thanks to the use of LC-MS/MS, which typically outperforms other analytical methods for drug quantification.

A total of 2’332 5-FU concentrations, measured in 726 individuals either under continuous infusion alone or preceded by a bolus, were used for the present analysis. Only 14 concentrations were below the LLOQ and thus removed from the analysis.

**Table 2:** Characteristics of the populations included in the IPD-MBMA

	SID 1	SID 2	SID 3
Number of measures	159	323	1850
Number of patients	21	30	675
Men - Women	14 − 7	20 − 10	388 − 287
Age (median (range)), years	65 (40–79)	63 (31–82)	69 (24–90)
Weight (median (range)), kg	68 (48–97)	76 (58–98)	67 (32–126)
Height (median (range)), cm	168 (149–188)	171 (144–194)	170 (150–191)
Body Surface Area (median (range)), m^2^	1.7 (1.5-2.0)	1.9 (1.6–2.2)	1.8 (1.2–2.5)
Lean body mass (median (range)), kg, using Janmahasatian formula [32]	50 (34–70)	56 (38–69)	49 (27–79)
Estimated glomerular filtration rate eGFR (median (range)), mL/min/1.73m^2^, using CKD-EPI 2021, capped at 140 mL/min/1.73m^2^	91 (27–115)	99 (49–120)	98 (2-133)
Chemotherapy regimen (number of patients)	LV5FU2 (21)	FOLFOX† (20) FOLFIRI† (6) 5FU† (4) †: + bevacizumab (9)	NA
Disease stage (number of patients)	NA	I-II (3) III (13) IV (14)	NA
Liver metastasis (number of patients)	Yes (16) ≤ 25% (9) > 25% to ≤ 50% (5) < 50% to ≤ 75% (2) No (21)	Yes (11) No (19)	NA
WHO/ECOG performance score (number of patients)	0 (1) 1 (12) 2 (8)	0 (11) 1 (17) 2 (2)	NA
Slow DPD metabolizers (% (number of patients))	52%, *n* = 11	63%, *n* = 19	*n* = 44, missing data for 325 individuals
Analytical method used to quantify 5-FU (LLOQ)	HPLC-UV (0.05 mg/L) [30]	LC-MS/MS (0.013 mg/L) [33]	LC-UV (0.03 mg/L) [18]

Datasets from Terret et al. (2002), Mueller et al. (2013) and the Dijon Clinical Cancer Center are referred to as SID1, SID2 and SID3, respectively. The demographic and clinical data is described with harmonized units

LV5FU2 = folinic acid, fluorouracil ; FOLFOX = folinic acid, fluorouracil, oxaliplatin ; FOLFIRI = folinic acid, fluorouracil, irinotecan; FOLFORINOX = folinic acid, fluorouracil, irinotecan, oxaliplatin

### Base and final pharmacokinetic model development

During model development, several possibilities regarding the number of compartments and the elimination kinetics were tested. For one-compartment models, mixed elimination (i.e., combined linear and Michaelis-Menten) and strictly non-linear elimination significantly improved the model compared to only linear elimination (ΔBIC = -603 and ΔBIC = -668, respectively). Adding a second compartment further improved the fit relative to the corresponding one-compartment models (mixed ΔBIC = -363, non-linear ΔBIC = -197). Ultimately, a mixed elimination model was retained as it outperformed a strictly non-linear model with two compartments (ΔOFV = -116).

After insertion/removal of IIV parameters in multiple combinations, IIV was kept on the drug elimination parameters V_max_ and CL (ΔOFV = -216 and ΔOFV = -159, respectively), as well as on V_1_ and V_2_ (ΔOFV = -74 and ΔOFV = -5, respectively). IIV on K_m_ was not retained for physiological plausibility, while IIV on inter-compartmental clearance (Q) did not reach statistical significance (ΔOFV = -1.9). Finally, the IIV of V_2_ was entirely explained by an allometric BW effect and was therefore fixed to 0%. One patient of the original SID2 dataset was excluded from this analysis as the associated η_V1_ value was clearly an unexplained outlier.

The residual variability was expressed using a conditional error model, with a grouped proportional error model for SID1 and SID2 and a separate mixed error model for SID3.

The significant covariates retained in the final model after stepwise selection procedure (detailed in Table S2 and Figure S2) are sex and age on V_max_, as well as BSA on CL. Clinically, this translates into an 18% increase in the V_max_ of men compared to women of the same age, and a 17% decrease in V_max_ for an 80-year-old patient compared to a 40-year-old of the same sex. Cumulatively, an 80-year-old woman has a 30% lower V_max_ than a 40-year-old man. A BSA of 1.5 m^2^ results in a 53% lower linear clearance CL value than a 2.0 m^2^ BSA.

**Table 3:** Final popPK model parameters with their bootstrap evaluations

PK parameter	Estimate (%RSE)	Bootstrap median (CI _95%_)
V_max_ (mg/h)	429 (22%)	388 (253, 490)
θ_Male_	0.183 (18%)	0.182 (0.116, 0.254)
θ_AGE_	-0.289 (25%)	-0.299 (-0.409, -0.181)
IIV V_max_ (%CV)	25 (5%)	25 (22, 28)
K_m_ (mg/L)	2.34 (22%)	2.12 (1.38, 2.70)
CL (L/h)	52.6 (13%)	56.0 (46.4, 69.3)
θ_BSA_	2.61 (14%)	2.51 (1.91, 3.15)
IIV CL (%CV)	34 (15%)	32 (26, 39)
V_1_ (L)	9.21 (24%)	9.72 (6.10, 19.8)
IIV V_1_ (%CV)	82 (28%)	76 (35, 131)
Q (L/h)	125 (19%)	119 (32.2, 157)
V_2_ (L)	14.6 (11%)	13.9 (7.39, 16.5)
θ_BW_	1, fixed	
σ_prop_ SID1 and SID2 (%)	24 (3%)	23 (21, 27)
σ_prop_ SID3 (%)	22 (2%)	22 (20, 25)
σ_add_ SID3 (mg/L)	0.0407 (4%)	0.0402 (0.0013, 0.0524)

Final model equations:

$$TV{V}_{max}={V}_{max} \cdot (1+\theta_{AGE}\cdot {\frac{AGE-60}{60}})\cdot(1+\theta_{Male}\cdot{SEX})$$

$$TVCL=CL\cdot{\left(\frac{BSA}{1.7}\right)}^{{\theta}_{BSA}}$$

$$TV{V}_{2}={V}_{2}\cdot{\left(\frac{BW}{70}\right)}^{{\theta}_{BW}}$$

Typical values TV of Maximum velocity V_max_, Michaelis-Menten constant K_m_, linear clearance CL, central and peripheral volumes of distribution V_1_ and V_2_ respectively, intercompartmental clearance Q. Effect of body-surface area (BSA), age (AGE), sex (men = 1, women = 0) and bodyweight (BW) covariates are reported as θ. Proportional (prop) and additive (add) errors are shown as σ. Relative standard errors (RSE) are reported as percentages. SID1, SID2 and SID3 refer to the datasets from Terret (2000), Mueller (2013) and the Dijon Clinical Cancer Center, respectively. Bootstrap median values are reported with 95% percentile confidence intervals (CI _95%_).

**Fig. 1:**
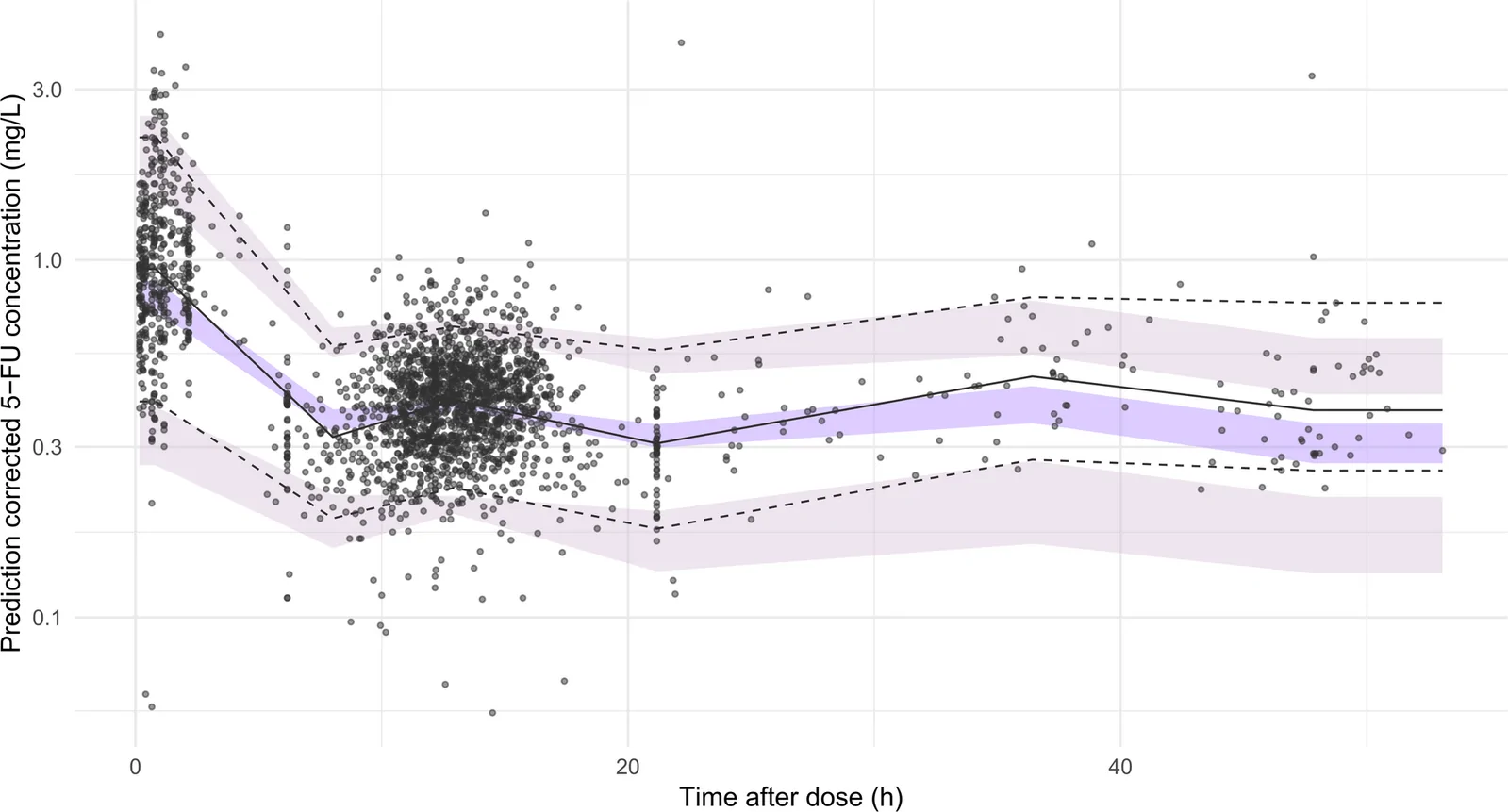
Prediction-corrected visual predictive check (pcVPC) for the final population PK model of 5-fluorouracil. The black points represent observed concentrations corrected by population predictions, plotted against time after dose. The solid black line represents the median of the observed data, and the dashed lines show the 5th and 95th percentiles. The dark purple shaded area represents the predicted 95% confidence interval for the median and the pink shaded area are the 2.5% and 97.5% percentiles

The shrinkage on V_max_, CL, and V_1_ amounted to 26%, 57% and 77%, respectively. Table 3 details the final model parameters together with their bootstrap evaluations (success rate > 95%). Differences between the model estimates and the bootstrap results all fall within ± 10%, and the estimates lie within the corresponding CI_95%_. Additionally, the pcVPC (Fig. 1) indicates that the model appropriately describes the central trend and variability in the data, with more misspecification at later time points due to the lack of data points in this area.

### Lean body mass and 5-FU disposition

Lean body mass appeared to be significant in univariate tests (Table S2) on the base popPK model of 5-FU on both V_max_ (ΔOFV = -166) and CL (ΔOFV = -141). After stepwise forward insertion/backward deletion steps, an alternative covariate popPK model was derived from our base model with significant impact of LBM and age on V_max_, as well as BSA effect on CL. Details on this alternative model (i.e., parameter estimates, bootstrap results, and pc-VPC) can be found in the supplementary material (Table S3 and Figure S5).

In our main model, the LBM component is carried through the sex. Indeed, LBM is estimated using the Janmahasatian’s formula, which includes sex, BW, and HT. A tight relationship between the two covariates was therefore expected.

### Simulations

**Fig. 2:**
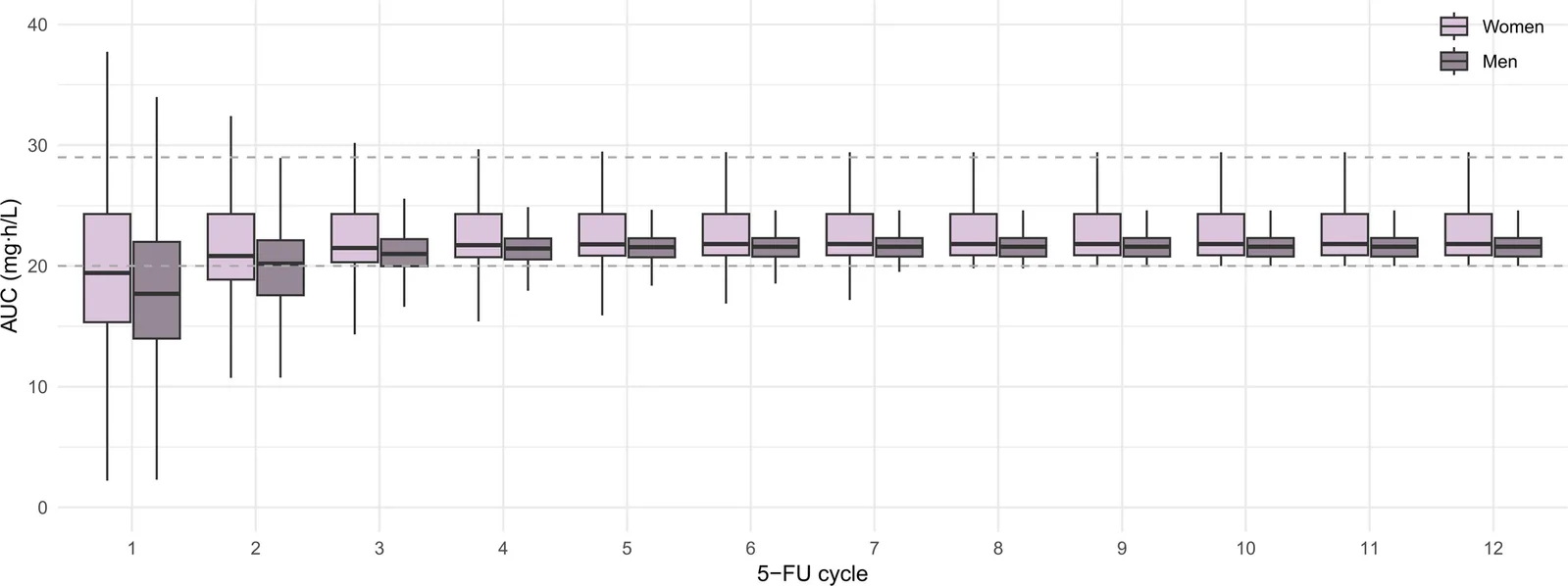
Simulated 5-FU cycles for target therapeutic range attainment. A virtual population of 30’000 patients in total received a bolus of 400 mg/m2 over 10 min, followed by a continuous infusion of 2’400 mg/m2 over 46 h, 30 min after the start of the bolus. AUCs were calculated using the concentration simulated 24 h after the start of the continuous infusion, multiplied by 46 h. Dosage was adapted from the AUC calculated at cycle n for cycle n + 1 based on the currently proposed dosage adjustment algorithm [11]

Figure 2 shows the simulation results on 30’000 virtual patients per cycle, per sex, using the classic dose adaptation grid.

During the first cycle, less than half of the population attained the target AUC of 20–29 mg∙h/L. At the second cycle, after one dosage adjustment, 60% and 51% of women and men, respectively, were within the target range. From the fifth cycle onwards, more than 95% of women and men reached the target exposure.

**Fig. 3:**
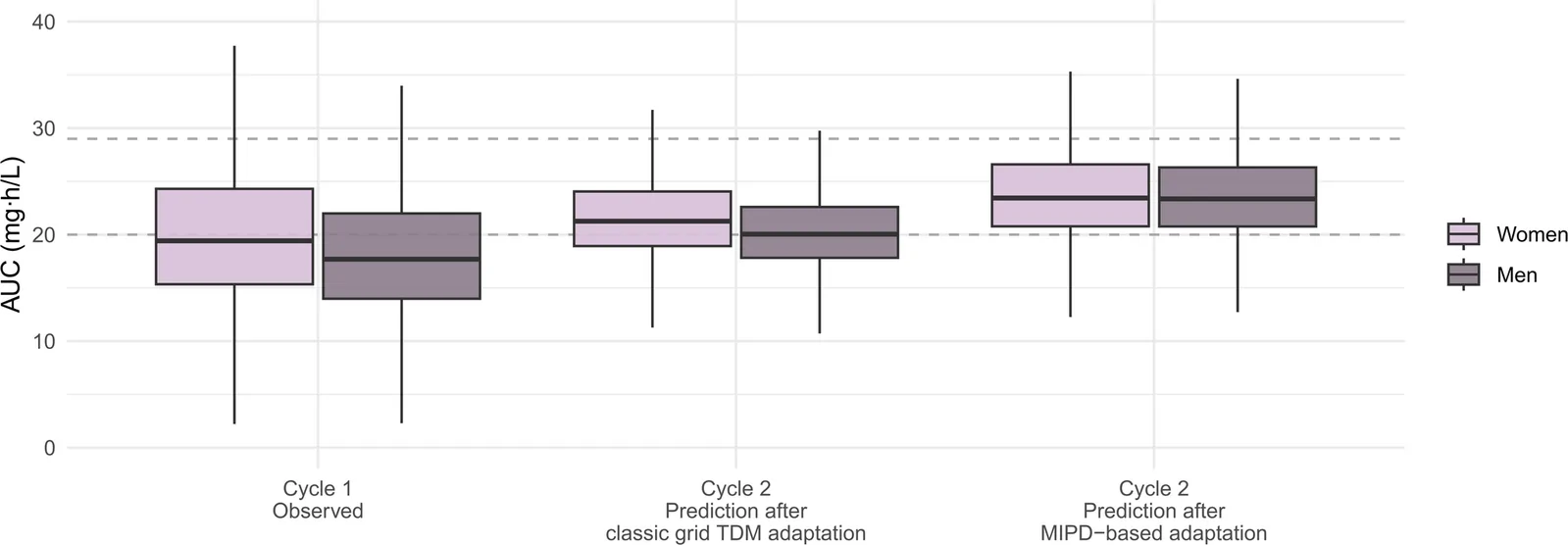
Simulated classic TDM approach versus MIPD-based virtual TDM approach. A virtual population of 30’000 patients in total received a bolus of 400 mg/m^2^ over 10 min, followed by a continuous infusion of 2’400 mg/m^2^ over 46 h, 30 min after the start of the bolus. For the observed cycle 1, AUCs were calculated using the concentration simulated 24 h after the start of the continuous infusion, multiplied by 46 h. For the prediction of cycle 2 after classic TDM adaptation, dosage was adapted from the AUC calculated at cycle 1 based on the classic dose adjustment grid [11]. For the prediction of cycle 2 after MIPD-based adaptation, individual PK parameters using a single TDM point of the observed cycle 1 were obtained, and used to predict individual PK curves and guide dose adaptation

Results of our simulated MIPD approach versus traditional TDM are shown in Fig. 3.

Based on our virtual classic TDM approach, predicted target attainment at cycle 2 was 59% and 49% for women and men, respectively. For the MIPD scenario, 68% of women and 70% of men were predicted to fall within optimal exposure after prediction-based dose adaptation. This analysis therefore anticipates a higher target attainment using MIPD-based adjustment over traditional TDM. A comparison of the MIPD-derived dose adjustment factor as a function of IPRED_24.5 h_ compared to the grid-based dose adaptation factor in the context of a 46-hour infusion can be found in the supplementary material (Figure S6).

Finally, the presence of a bolus before the continuous infusion did not impact the resulting clinical AUC, and neither did the schedule nor the duration of the bolus (supplementary material Figure S7).

## Discussion

This study represents the first 5-FU model-based meta-analysis using individual patient data. To our knowledge, it combines the largest number of patients to date to elaborate a popPK model for 5-FU. Aggregating individual patient data from three different sources, our analysis reveals that a two-compartment model with mixed linear and Michaelis-Menten elimination best describes 5-FU disposition. This model is physiologically coherent, as 5-FU is processed through several pathways. Indeed, the DPD enzyme represents a saturable pathway, likely reflected in the Michaelis-Menten portion of the clearance [34]. On the other hand, a minor part (< 20%) of 5-FU is excreted unchanged in urine, in accordance with a linear component of the clearance. Together, these factors help to explain that a mixed elimination best fits the observations. Available literature is relatively heterogeneous regarding 5-FU disposition: some studies obtain better results with either one [35] or two [36] compartments, and with strictly linear [37] or non-linear elimination [38]. This highlights the fact that, as models are built to fit the studied population, different study characteristics and sampling schedules inform the models in different ways: a meta-analysis approach integrating these disparities and capturing multiple sampling scenarios helps to characterize the drug’s PK more globally.

It is important to note that despite the relatively high shrinkage observed on CL and V_1_, the use of the stepwise methodology based on the likelihood ratio test rather than individual posterior estimates to explore covariate effect ensures the reliability of the identified covariate-parameter relationships [39]. The significant covariates retained on 5-FU elimination were BSA, age, and sex or LBM, per our two analyses. Firstly, the effect of age on elimination parameters in an adult-only population was previously documented for 5-FU and is explainable by decreasing metabolic capacity due to reduced hepatic perfusion for example [40]. Then, the sizeable impact of sex on 5-FU PK is well known and has been extensively discussed throughout the literature [41]. In our final model, if we isolate the effect of sex on drug elimination, men show an 18% higher V_max_. Sex-based differences in physiology may explain this lower V_max_ in women, who have less lean tissue mass and different metabolic capacity than men [42, 43], as illustrated by our alternative model, where the effect of sex is captured by an LBM effect. For the linear part of the clearance, BSA was the only significant covariate retained, and it is worth noting that although no significant effect of eGFR was found in our analysis, a recently published study found a stronger correlation between 5-FU clearance and eGFR than with uracil, although the latter is commonly used for predicting delayed 5-FU elimination [44]. Information on the impact of renal function on 5-FU disposition is scarce yet deserves more consideration in light of growing evidence of its potential impact. Overall, risks of underexposure in men and increased toxicity in women raise the question of sex-adapted dosing strategies [41]. Clinical trials with sex-adapted doses should be considered in order to provide better *a priori*-dosing strategies beyond BSA-dosing [45]. 

BSA is a significant factor correlated with 5-FU clearance in our analysis, and it is also the current basis for 5-FU dosing. Its significant association with 5-FU elimination is therefore justified; however, dosing based on this historical metric is debated [4–6]. On the other hand, LBM might be a clinically relevant parameter in oncology, as chemotherapeutic agents dispose predominantly within lean tissue, imperfectly reflected in BSA or total body weight [8]. Consequently, some patients with high bodyfat but low LBM may be overdosed, heightening the risk of toxicity [46, 47], while others with high LBM but low bodyfat may be underdosed. In our parallel analysis, we uncovered a significant impact of LBM on 5-FU elimination: as LBM increases, V_max_ increases, resulting in higher metabolic capacity. The first model presented, ignoring LBM as covariate, favoured a simpler metric, immune to misestimation: sex. On the other hand, the LBM formula employed in our second model is derived from three parameters: sex, BW, and HT. Therefore, we tend to favour the first model, as it comprises simpler body metrics. Indeed, LBM has its own limitations for the estimation of true FFM. When no measure of the latter is available, one of the various formulas for LBM available in the literature can be used. In our study, we chose the Janmahasatian’s formula [29], particularly suited to the age of our population [32]. A model involving simpler metrics seems, therefore, less prone to uncertainty than a model implementing a rather sophisticated LBM formula without a clear advantage. However, favouring true FFM measures seems appealing in the future for tailoring therapies as imagery-based algorithms become available, taking advantage of radiologic investigations routinely performed in patients. This opens the discussion for FFM-based dosing, called upon to replace the debated BSA-based dosing.

Grouping data from multiple studies leads to inevitable concessions regarding the information available and imposes limitations to such studies. Indeed, the IPD-MBMA approach implies losing information if some characteristics are scarcely reported across studies, e.g., imposing to convert continuous variables into categories, such as with the DPD metabolic profile in our case, or not being able to investigate a potential covariate such as for example the infusion system (elastomeric or electronic). The classic clinical metrics (sex, weight, height, age, etc.) are however quite systematically reported, fortunately, enabling the identification of relevant covariates. Further underlying differences between datasets, such as those related to analytical technique or patient management, can be simply described using separate error models for each study, as shown in our popPK analysis.

According to our simulations, target AUC attainment at the first cycle of 5-FU is reached in less than half of the patients (36% of women and 29% of men), indicating that without personalized dosage adjustment, there is a high chance for under- or over-dosing patients. With the implementation of a rather simple TDM routine, the next cycle showed an increase of  24% and 22% in target attainment for women and men, respectively. Finally, after 4 rounds of adjustment, i.e. at the fifth 5-FU cycle, more than 95% of patients were within optimal systemic exposure. It might seem surprising that as many as 4 cycles of adjustments are needed; this may actually be due to the relatively imprecise dose adjustment rules, together with the inclusion of random residual errors in our simulations, which actually best mimic the real-world conditions of TDM practice and 5-FU variability, e.g. circadian variation in metabolism. On this matter, one limitation of the present study was the inability to model the circadian rhythm due to the nature of the data, as some of it contained only relative time after dose and not true schedule. This simulation strongly underlines the importance of 5-FU TDM as per current recommendations, with an elementary, easy-to-implement dose adjustment algorithm [11]. In a prospective clinical trial looking at target attainment (20–29 mg∙h/L), dose adjustment based on the same adaptation algorithm showed a maximum of 65% of patients within target range at the third cycle [48]. Another study looked at target attainment within 20–25 mg∙h/L and showed that BSA-dosing significantly underdoses patients, while by the fourth cycle, TDM adjustments allowed 47% of patients to reach the target exposure [49]. Our simulations align with the conclusions that TDM-guided dose adjustment can drastically improve target attainment in a great proportion of patient. Yet, TDM strategies could still be improved. Indeed, five cycles represent two months of treatment, during which exaggerated dosing may cause uncomfortable side effects such as nausea, vomiting, and the infamous “chemo brain” among others; on the other hand, underexposure (more frequent in men) might increase the risk of treatment failure. MIPD is therefore an area to explore, as it could provide both *a priori* and early *a posteriori* optimal estimates of drug exposure for target attainment. Indeed, model-based dose recommendations can integrate the significant covariates and already refine the decision on the initial dose to give to a patient. MIPD may then improve the precision of subsequent dose adaptations informed by TDM. Based on our virtual TDM simulation results, roughly 70% of patients are predicted to be adequately exposed after one MIPD-guided adjustment, against 59% and 49% of women and men, respectively, for the traditional grid-guided dose adjustment. The virtual TDM approach used in this study is a first step urgently calling for a more extensive validation. Indeed, a non-linear dose adjustment factor *f* was derived using *post hoc* individual PK parameters, illustrating MIPD feasibility while taking into account the non-linearity of 5-FU PK: this mimics the maximum *a posteriori* empirical Bayes estimates (MAP EBEs) step. True MIPD is, however, not limited to this: the dose can then be optimized to maximize probability of target attainment (PTA), which means the right dose is chosen in order to maximize the chance for patients to reach an AUC of 20–29 mg∙h/L. In our *in silico* experiment, we chose a single median point of 24 mg∙h/L as the best optimal target, for feasibility reasons. This proof-of-concept experiment, although minimally simplified, motivates the need for real-life clinical trials using MIPD to definitely assess its benefits over, or alongside, traditional TDM. The grid adaptation algorithm currently used is already a useful decision tool, but we show that an MIPD-based decision would typically lead to more drastic dose increases or decreases. In MIPD in general, the clinician’s expertise remains always required at final step, to mitigate risks and benefits of aggressive dose changes. Thus, the traditional dose adaptation rid might be considered as already integrating an amount of clinical cautiousness, possibly mitigating the PTA improvement to expect from MIPD in a real-world comparative trial. A recently published review [50] establishes the current state of MIPD versus traditional TDM in oncology, showing a disappointing lack of data for 5-FU, especially since many models are available. These preliminary *in silico* data may encourage the pursue of clinical studies in that direction.

We also evaluated the impact of the initial bolus injection, including the case where no bolus is given, on AUC calculation in a clinical setting (i.e. multiplying a late concentration by infusion duration). Our simulation predicts that no bias is expected in current practice, yet a real-life study remains needed to confirm this. Since the bolus portion is not considered in the current computation of AUC, and our simulations show no differences in the evaluated exposure, the question of the utility of the bolus remains open, as already discussed in the literature [51]. Indeed, the bolus is often left out because patients cannot tolerate it due to toxicity; some studies even show that eliminating the bolus seems not to alter treatment efficacy [52]. To our knowledge, literature data on the actual impact of the bolus on resulting AUC is still lacking: the only study evaluating the impact of a bolus on 5-FU exposure [53] uses oral tegafur, a prodrug of 5-FU.

From a methodological perspective, this study illustrates the advantages of benefiting from data sharing after publication to enable broader research. While published articles can serve as a key source for summary-level meta-analyses, less-accessible raw data offer more depth for individual-patient meta-analyses, which can in turn yield more precise conclusions better benefiting clinical practice. In addition, meta-level analyses can highlight gaps in current knowledge, guiding future studies in focused directions. Difficulties in retrieving relevant raw data from the literature should not be overlooked [54]. Firstly, contacting authors is not always trivial, as some may have retired or changed institution and others may neglect data-sharing requests for all kinds of reasons. Reluctance to directly share data with unknown people is quite common and understandable. The time needed to access a large number of datasets is consequential, yet guidelines providing valuable methods for doing so are emerging [54], paving the way for a more generalized utilization of all published knowledge.

Overall, this study points to a direct next step, which is prospective evaluation. The added value of MBMA versus model selection/averaging [55] to derive optimal MIPD strategies should be evaluated. Above all, however, the goal is to move beyond predictive performance and test real-life clinical benefits, i.e. whether model-guided dosing improves patient outcomes compared to traditional TDM in prospective interventional trials. One concern is that such trials might require sizeable patient samples to ensure sufficient statistical power. Even once validation is granted, implementation will be limited by logistics: unequal access to 5-FU assays, despite LC-MS/MS methods [56] and commercial solutions such as My5-FU^®^, and the need to raise oncologists’ awareness and train teams, as TDM remains rather uncommon in oncology. Experts are already advocating for both strengthened TDM practice [11] and easier measurement [57, 58], and these ongoing efforts are key to further refining 5-FU treatment.

## Conclusion

Aggregating available PK data from the literature allows for building well-informed models that capture a fairly global patient population with diverse dosing and sampling scenarios for a given drug. Using stratified error models allows for easy capture of differences in residual variability, as data come from multiple sources. A global effort to share raw data from published studies is needed to join forces, build models to best summarize available information, highlight remaining knowledge gaps, and guide future studies in a pertinent direction. As widely acknowledged in the literature, 5-FU is a drug for which TDM is required for safe and effective use. Dosage tailoring using BSA still leaves substantial inter-patient variability in terms of drug exposure. Still, LBM-based dosing deserves consideration since it might better take into account sex-related metabolic differences, of importance in the context of 5-FU. Prospective clinical studies on the impact of both *a priori* model-informed dosing and MIPD-assisted TDM are warranted, motivated by our preliminary *in silico* results. There remains room for improvement in the precision dosing of 5-FU and personalized patient care.

## Supplementary Information

Below is the link to the electronic supplementary material.

Supplementary Material 1

## Data Availability

Data are available from the corresponding author upon reasonable request, subject to data-sharing agreements and applicable approvals.
