# Supplementary material for: Model-based meta-analysis of individual patient data for the characterization of intravenous 5-fluorouracil population pharmacokinetics
Source: Cancer Chemother Pharmacol. 2026 Jun 12;96(1):63. doi: 10.1007/s00280-026-04885-x (PMC13661680; doi:10.1007/s00280-026-04885-x)
Supplement: Supplementary file 1 [file 280_2026_4885_MOESM1_ESM.docx]

**Supplementary Material**

**—**

**Model-based meta-analysis of individual patient data for the characterization of intravenous 5-fluorouracil population pharmacokinetics**

Myriam Briki (1,2)*, Paul Thoueille (1,3), Markus Joerger (4), Etienne Chatelut (5), Marylise Sterle (6), Antonin Schmitt (6), Anna Dorothea Wagner (7), Thierry Buclin (1), Sandro Carrara (2), Monia Guidi (1,8)

(1) Service of Clinical Pharmacology, Department of Medicine, Lausanne University Hospital and University of Lausanne, Lausanne, Switzerland.

(2) Bio/CMOS Interfaces Laboratory, Department of Engineering, Swiss Federal Institute of Technology in Lausanne - EPFL, Switzerland.

(3) Laboratory of Clinical Pharmacology, Department of Laboratory Medicine and Pathology, Lausanne University Hospital and University of Lausanne, Lausanne, Switzerland.

(4) Department of Oncology and Hematology, Cantonal Hospital St. Gallen, St. Gallen, Switzerland.

(5) University of Toulouse, Oncopole Claudius Regaud, IUCT–Oncopole, INSERM UMR1037, Toulouse, France.

(6) Université Bourgogne Europe, Centre Georges-François Leclerc, Service Pharmacie, INSERM, CTM UMR 1231, TIRECS, Dijon, France.

(7) Department of Oncology, Division of Medical Oncology, Lausanne University Hospital and University of Lausanne, Lausanne, Switzerland.

(8) Centre for Research and Innovation in Clinical Pharmaceutical Sciences, Lausanne University Hospital and University of Lausanne, Lausanne, Switzerland.

*Corresponding author: Myriam Briki [myriam.briki@epfl.ch](mailto:myriam.briki@epfl.ch)

Table of contents

[1.	Data specifications	2](#_Toc220661667)

[2.	Covariate analysis	5](#_Toc220661668)

[3.	Visual inspection of the final model	7](#_Toc220661669)

[4.	Alternative popPK model with Lean Body Mass (LBM) as a covariate	8](#_Toc220661670)

[5.	Virtual TDM approach	9](#_Toc220661671)

[6.	Impact of bolus schedule on AUC	11](#_Toc220661672)

[7.	NONMEM code	11](#_Toc220661673)

1. Data specifications

Table S1 - **Data processing details.** This table lists the columns of the final dataset and explains how information from the original datasets was either changed or left unchanged (◊) to combine them.

|  | Identifier | Terret et al. 2001 | Mueller et al. 2013 | Dijon-Bourgogne Hospital |
| --- | --- | --- | --- | --- |
| Study ID | SID | 1 | 2 | 3 |
| Patient ID | ID | ◊ | ID + 22 | ID + 22 + 32 |
| Time after dose | TAD | ◊ | ◊ | Calculated using real times |
| Dose (mg) | AMT | ◊ | Converted from mmol to mg (MW = 130.078) | ◊ |
| Rate of infusion (mg/h) | RATE | ◊ | Converted from mmol/h to mg/h (MW = 130.078) | ◊ |
| Duration of infusion (h) | TK0 | AMT/RATE | AMT/RATE | ◊ |
| Compartment | CMT | 1 | Discard CMT = 2 (metabolite) | 1 |
| Event ID | EVID | ◊ | ◊ | 1 for first cycle, 4 for every new cycle |
| Observations (mg/L) | DV | ◊ | Converted from ln(µmol/L) to mg/L | Converted from µmol/L to mg/L |
| Censor observation | MDV | ◊ | ◊ | 1 if:   - DV < LOQ - TAD = 0 - Aberrant DV after end of infusion (n = 6): DV > 0.05 mg/L at least 2h after end of infusion - Aberrant DV after the start of continuous infusion |
| Sex (male = 1, female = 0) | SEX | Switched 1 to male and 0 to female | ◊ | ◊ |
| Age (years) | AGE | ◊ | ◊ | Converted from post-menstrual age in weeks to years (PMAW ∙ 7 / 365) |
| Weight (kg) | WT | ◊ | ◊ | ◊ |
| DPD metabolizer status (slow = 1, else = 0) | DPD_SLOW | 1 if DPD activity < 150 pmol/min/mg | 1 if uracil ≥ 16 ng/mL | 1 if uracil ≥ 16 ng/mL |
| Creatinine clearance (mL/min)  → Cockcroft-Gault^1^ (CG) formula, capped at 140 mL/min to adjust for the overestimation caused by low lean body mass, as done in SID2 | CLCR | Available: serum creatinine (µM), converted from µM to mg/dL (factor: 0.011)  Applied CG formula | ◊ | Available: serum creatinine (mg/dL)  Applied CG formula |
| Body surface area (m^2^) | BSA | ◊ | ◊ | √(HT ∙ WT/3600) |
| Height (cm) | HT | ◊ | BSA^2^ ∙ 3600/WT | ◊ |
| Glomerular filtration rate (mL/min/1.73 m^2^)  → CKD-EPI 2021 serum creatinine formula (without race), capped at 140 mL/min/1.73 m^2^ to adjust for the overestimation caused by low lean body mass | GFR | Available: serum creatinine (µM), converted from µM to mg/dL (factor: 0.011)  Applied CKD-EPI 2021 formula | Available: CLCR with CG  Extracted serum creatinine concentration from CLCR using CG  Applied CKD-EPI 2021 formula | Available: serum creatinine (mg/dL)  Applied CKD-EPI 2021 formula |
| Lean body mass (kg)  → Harmonized using Janmahasatian formula^2,3^ | LBM | Applied formula using provided WT, HT, and SEX | Applied formula using provided WT, HT derived from BSA, and SEX | Applied formula using provided WT, HT, and SEX |

**Fig.S1 –** **Observed 5-FU concentrations over time.** Concentrations of 5-FU are represented, stratified by studies (SID). The figure shows heterogeneity of concentrations with respect to time.

2. Covariate analysis

**Table S2 -** **Summary of univariate covariate analysis.**

| PK parameter | Covariate | ΔOFV  (p-value: * <0.05 ; ** < 0.01 ; *** < 0.001) |
| --- | --- | --- |
| V_max_ | SEX | -95.3 (***) |
|  | BW | -120 (***) |
|  | BSA | -153 (***) |
|  | LBM | -166 (***), see alternative model |
|  | AGE | -17.8 (***) |
|  | HT | -115 (***) |
|  | DPD_SLOW | 0.18 |
|  |  |  |
| CL | SEX | -85.4 (***) |
|  | BW | -138 (***) |
|  | BSA | -159 (***) |
|  | eGFR linear / sigmoid | -1.71 / -1.34 |
|  | LBM | -141 (***), see alternative model |
|  | AGE | -13.6 (***) |
|  | HT | -67.1 (***) |
|  | DPD_SLOW | -0.85 |
|  |  |  |
| V_1_ | SEX | -3.63 |
|  | BW | -4.89 (*) |
|  | BSA | -1.73 |
|  | LBM | -3.36 |
|  | AGE | -2.95 |
|  | HT | -3.43 |
|  | DPD_SLOW | -0.44 |
|  |  |  |

**Fig.S2 – Forward insertion and backward deletion process.** The full covariate selection step is detailed for **A.** the main final model and **B.** the alternative final model with LBM.

**Fig.S3 -** **Graphs of significant covariates distribution.** Distribution of body surface area (BSA), age (AGE), lean body mass (LBM) and body weight (BW) per sex and per study (SID). SID1, SID2 and SID3 refer to the datasets from Terret et al. (2000), Mueller et al. (2013) and the Dijon-Bourgogne Hospital, respectively.

3. Visual inspection of the final model

**Fig.S4 –** **Goodness of fit diagnostic plots of the final model presented in the main article.** Obtained data is represented with stratification per study-identifier (SID); SID1, SID2 and SID3 refer to the datasets from Terret (2000), Mueller (2013) and the Dijon-Bourgogne Hospital, respectively. The correspondence of **A.** individual predictions and **B.** population prediction with observed concentrations is represented with respect to the dotted identity line. Conditional weighted residuals (CWRES) are plotted against **C.** population predictions (PRED) and **D.** time. Overall, the CWRES are centered around zero, with no clear trend or systematic deviation across the range of predicted values. Although there is some scatter—especially at lower predicted concentrations and time points where most of the data points cluster—the distribution does not suggest any major bias or concentration-dependent error. This indicates that the model’s predictions are reasonably aligned with observed data throughout the concentration range and the studied time.

4. Alternative popPK model with Lean Body Mass (LBM) as a covariate

**Table S3 –** **Alternative final model with LBM.**

| PK parameter | Estimate (%RSE) | Bootstrap median [PCI 95%] |
| --- | --- | --- |
| V_max_ (mg/h) | 411 (23%) | 419 [271, 515] |
| θ_LBM_ | 0.484 (18%) | 0.491 [0.311, 0.690] |
| θ_AGE_ | -0.292 (25%) | -0.292 [-0.409, -0.168] |
| IIV V_max_ (%CV) | 26 (5%) | 26 [23, 29] |
| K_m_ (mg/L) | 1.89 (24%) | 1.91 [1.22, 2.42] |
| CL (L/h) | 59.2 (11%) | 59.5 [49.5, 71.6] |
| θ_BSA_ | 1.62 (25%) | 1.56 [0.772, 2.38] |
| IIV CL (%CV) | 31 (15%) | 31 [24, 37] |
| V_1_ (L) | 9.58 (21%) | 9.86 [5.87, 19.4] |
| IIV V_1_ (%CV) | 75 (25%) | 70 [31, 124] |
| Q (L/h) | 112 (20%) | 107 [30.9, 150] |
| V_2_ (L) | 13.6 (12%) | 13.2 [7.39, 16.1] |
| θ_BW_ | 1, fixed |  |
| σ_prop_ SID1 and SID2 | 23.8 (3%) | 23.5 [20.7, 26.6] |
| σ_prop_ SID3 | 21.7 (2%) | 21.9 [19.7, 24.6] |
| σ_add_ SID3 (mg/L) | 0.0405 (4%) | 0.0402 [0.001254, 0.0520] |

Model equations:

$TVV_{max}=V_{max} \cdot\left( 1+\theta_{AGE}\cdot\frac{AGE-60}{60} \right)\cdot\left( \frac{LBM}{55} \right)^{\theta_{LBM}}$,

$TVCL=CL \cdot\left( \frac{BSA}{1.7} \right)^{\theta_{BSA}}$,

$TVV_{2}=V_{2}\cdot\left( \frac{BW}{70} \right)^{\theta_{BW}}$ .

Typical values TV of Maximum velocity V_max_, Michaelis-Menten constant K_m_, linear clearance CL, central and peripheral volumes of distribution V_1_ and V_2_ respectively, intercompartmental clearance Q. Effect of lean body mass (LBM), body-surface area (BSA), sex and bodyweight (BW) covariates are reported as θ. Proportional (prop) and additive (add) errors are shown as σ. Relative standard errors (RSE) are reported as percentages. SID1, SID2 and SID3 refer to the datasets from Terret (2000), Mueller (2013) and the Dijon-Bourgogne Hospital, respectively. Bootstrap median values are reported with 95% percentile confidence intervals (CI 95%).

**Fig.S5** – **Prediction corrected visual predictive check (pcVPC) for the alternative final population PK model of 5-fluorouracil including LBM as a covariate**. The black points represent observed concentrations corrected by population predictions, plotted against time after dose. The solid black line represents the median of the observed data, and the dashed lines show the 5th and 95th percentiles. The dark purple shaded area represents the predicted 95% confidence interval for the median.

5. Virtual TDM approach

$$\frac{dA}{dt}=Rate-CL\cdot C-\frac{V_{max}\cdot C}{K_{m}+C}$$

At steady state, $\frac{dA}{dt}=0$ :

$$\frac{dA}{dt}=0=Rate-CL\cdot C-\frac{V_{max}\cdot C}{K_{m}+C}$$

$$Rate=CL\cdot C+\frac{V_{max}\cdot C}{K_{m}+C}$$

From this, we rewrite the rate as a function of the concentration and obtain a dose adaptation factor *f* as such:

$$f=\frac{Rate(C_{target})}{Rate(C_{pred})}=\frac{{CL}_{i}\cdot C_{target}+\frac{V_{max, i}\cdot C_{target}}{K_{m, i}+C_{target}}}{{CL}_{i}\cdot C_{pred}+\frac{V_{max,i}\cdot C_{pred}}{K_{m,i}+C_{pred}}}$$

We have the following equivalences:

$$Rate=\frac{Dose}{Time of infusion}$$

$$AUC=C\cdot Time of infusion$$

Therefore, since the time of infusion is constant in our context, we can rewrite *f* as the factor to apply to the next given dose:

$$f=\frac{{CL}_{i}\cdot C_{target}+\frac{V_{max, i}\cdot C_{target}}{K_{m,i}+C_{target}}}{{CL}_{i}\cdot C_{pred}+\frac{V_{max, i}\cdot C_{pred}}{K_{m,i}+C_{pred}}}$$

Where CL_i_, V_max,i_ and K_m,i_ are individual PK parameters estimated *post hoc*, C_target_ is AUC_target_/infusion time (here, 24 mg∙h∙L^-1^ / 46h), and C_pred_ is IPRED_24.5h_, or more generally, IPRED late enough during the infusion so that the concentrations are stabilized.

**Fig.S6 -** **Dose adaptation factors for classic grid TDM and MIPD-based adjustment rules for a 46-hour continuous infusion.** (A) Classic TDM grid-based dose multiplier as a function of the in silico observed 5-FU concentration at 24.5 h. (B) MIPD-based dose multiplier as a function of the 5-FU concentration predicted at 24.5 h (IPRED); the black curve uses typical population values of V_max_, CL and K_m_ from the final model (main manuscript), and points show individual simulated values. (C) Relationship between the in silico observed concentration at 24.5 h (cycle 1) and the MIPD-based dose multiplier computed from post hoc IPRED; points represent individuals, and the black curve shows the empirical trend. The green dashed line at y = 1 is shown to help visualize adaptations with respect to a no-change zone.

6. Impact of bolus schedule on AUC

**Fig.S7 -** **Bolus schedule impact on 5-FU AUC.** A virtual population of 15’000 women and 15’000 men was generated, and a 46h 2’400 mg/m² continuous infusion of 5-FU, alone or preceded by either a 10min 400 mg/m² bolus given 30min before the infusion or a 2min 400 mg/m² bolus given right before the infusion, was simulated on the whole virtual population for each scenario. AUC was calculated by multiplying the individual prediction (IPRED) 24h after the start of the continuous infusion by 46h.

7. NONMEM code

The NONMEM code corresponds to the main final model presented in the manuscript, while italic alternative code is for the final model containing LBM.

```
$PROBLEM PK

$INPUT SID ID TAD=TIME AMT RATE TK0 CMT EVID DV MDV SEX AGE WT DPD_SLOW CLCR BSA HT GFR LBM

$DATA SID_123_FINAL.csv IGNORE=#

$SUBROUTINES ADVAN13 TOL=3

$MODEL NCOMP=2
COMP=(CENTRAL)
COMP=(PERIPH)

$PK

REF_BW = 70
ADJ_BW = WT / REF_BW
IF(WT.LT.0) ADJ_BW = 1

REF_BSA = 1.7
ADJ_BSA = BSA / REF_BSA
IF(BSA.LT.0) ADJ_BSA = 1

REF_AGE = 60
ADJ_AGE = (AGE - REF_AGE) / REF_AGE
IF(AGE.LT.0) ADJ_AGE = 0

; REF_LBM = 55
; ADJ_LBM = LBM / REF_LBM
; IF(LBM.LT.0) ADJ_LBM = 1


TVVm = THETA(1)
Vm = TVVm * EXP(ETA(1)) * (1 + ADJ_AGE*THETA(12)) * (1 + SEX*THETA(13))
; Vm = TVVm * EXP(ETA(1)) * (1 + ADJ_AGE*THETA(12)) * (ADJ_LBM**THETA(13))

TVKm = THETA(2)
Km = TVKm * EXP(ETA(2))

TVCL = THETA(3)
CL = TVCL * EXP(ETA(3)) * (ADJ_BSA**THETA(11))

TVV1 = THETA(4)
V1  = TVV1 * EXP(ETA(4))

TVQ = THETA(5)
Q = TVQ * EXP(ETA(5))

TVV2 = THETA(6)
V2  = TVV2 * EXP(ETA(6)) * (ADJ_BW**THETA(10))

K12 = Q/V1
K21 = Q/V2

S1 = V1

$DES
CC = A(1)/V1
DADT(1) = ((-Vm*CC)/(Km+CC)) - K12*A(1) + K21*A(2) - (CL/V1) * A(1)
DADT(2) =  K12*A(1) - K21*A(2)

$ERROR

IPRED = A(1)/S1

W1 = SQRT(THETA(7)**2*IPRED**2)
W2 = SQRT(THETA(7)**2*IPRED**2)
W3 = SQRT(THETA(8)**2*IPRED**2 + THETA(9)**2)

IF (SID.EQ.1) THEN ; Proportional and additive error model for SID 1
  Y = IPRED + W1 * ERR(1)
ENDIF

IF (SID.EQ.2) THEN ; Proportional and additive error model for SID 2
  Y = IPRED + W2 * ERR(2)
ENDIF

IF (SID.EQ.3) THEN ; Proportional and additive error model for SID 3
  Y = IPRED + W3 * ERR(3)
ENDIF

$THETA
(0, 429) ; Vm
(0, 2.34) ; Km
(0, 52.6) ; CL
(0, 9.21)  ; V1
(0, 125) ; Q
(0, 14.6) ; V2
(0, 0.24) ; Prop SID1 + 2
(0, 0.22)  ; Prop SID3
(0, 0.04) ; Add SID3
(1) FIX ; V2 ~ BW
(2.61) ; CL ~ BSA
(-0.29) ; Vm ~ AGE
(0.18) ; Vm ~ SEX
; (0.48) ; Vm ~ LBM

$OMEGA
0.061 ; IIV Vm
0 FIX ; IIV Km
0.109 ; IIV CL
0.516 ; IIV V1
0 FIX ; IIV Q
0 FIX ; IIV V2

$SIGMA
1 FIX ; Residual error
1 FIX
1 FIX

$EST METHOD=1 LEVCENTER=0 INTERACTION MAXEVAL=9999 NOABORT SIGL=3 NSIG=1 PRINT=5 POSTHOC
$COV MATRIX=S

; Xpose
$TABLE SID ID TIME DV MDV EVID PRED IPRED CWRES CWRESI ONEHEADER NOPRINT FILE=sdtab
$TABLE SID ID Vm Km CL V1 Q V2 ETA1 ETA2 ETA3 ETA4 ETA5 ETA6 ONEHEADER NOPRINT FIRSTONLY FILE=patab
$TABLE SID ID Vm Km CL V1 Q V2 ETA1 ETA2 ETA3 ETA4 ETA5 ETA6 TIME AMT RATE TK0 CMT EVID DV MDV SEX AGE WT DPD_SLOW CLCR BSA HT GFR LBM ONEHEADER NOPRINT FILE=covtab
```

**References**

1	Cockcroft, D. W. & Gault, M. H. Prediction of creatinine clearance from serum creatinine. *Nephron* **16**, 31-41 (1976). <https://doi.org:10.1159/000180580>

2	Janmahasatian, S. *et al.* Quantification of lean bodyweight. *Clin Pharmacokinet* **44**, 1051-1065 (2005). <https://doi.org:10.2165/00003088-200544100-00004>

3	Puri, T. & Blake, G. M. Comparison of ten predictive equations for estimating lean body mass with dual-energy X-ray absorptiometry in older patients. *Br J Radiol* **95**, 20210378 (2022). <https://doi.org:10.1259/bjr.20210378>
